# Roles and therapeutic targeting of ceramide metabolism in cancer

**DOI:** 10.1016/j.molmet.2024.101936

**Published:** 2024-04-09

**Authors:** Narendra Wajapeyee, Teresa Chiyanne Beamon, Romi Gupta

**Affiliations:** 1 Department of Biochemistry and Molecular Genetics, University of Alabama at Birmingham, Birmingham, AL, 35233, USA; 2 O'Neal Comprehensive Cancer Center, University of Alabama at Birmingham, Birmingham, AL, 35233, USA

**Keywords:** Ceramide, Sphingolipids, Cancer therapy, Signaling, Nanoparticles, Nanoliposome

## Abstract

**Background:**

Ceramides are sphingolipids that act as signaling molecules involved in regulating cellular processes including apoptosis, proliferation, and metabolism. Deregulation of ceramide metabolism contributes to cancer development and progression. Therefore, regulation of ceramide levels in cancer cells is being explored as a new approach for cancer therapy.

**Scope of the review:**

This review discusses the multiple roles of ceramides in cancer cells and strategies to modulate ceramide levels for cancer therapy. Ceramides attenuate cell survival signaling and metabolic pathways, while activating apoptotic mechanisms, making them tumor-suppressive. Approaches to increase ceramide levels in cancer cells include using synthetic analogs, inhibiting ceramide degradation, and activating ceramide synthesis. We also highlight combination therapies such as use of ceramide modulators with chemotherapies, immunotherapies, apoptosis inducers, and anti-angiogenics, which offer synergistic antitumor effects. Additionally, we also describe ongoing clinical trials evaluating ceramide nanoliposomes and analogs. Finally, we discuss the challenges of these therapeutic approaches including the complexity of ceramide metabolism, targeted delivery, cancer heterogeneity, resistance mechanisms, and long-term safety.

**Major conclusions:**

Ceramide-based therapy is a potentially promising approach for cancer therapy. However, overcoming hurdles in pharmacokinetics, specificity, and resistance is needed to optimize its efficacy and safety. This requires comprehensive preclinical/clinical studies into ceramide signaling, formulations, and combination therapies. Ceramide modulation offers opportunities for developing novel cancer treatments, but a deeper understanding of ceramide biology is vital to advance its clinical applications.

## 1 Introduction

Ceramide metabolism involves a complex network of cellular processes including the synthesis, degradation, and interconversion of ceramide molecules, which play an important role in regulating ceramide levels and maintaining cellular homeostasis [[1], [2], [3]]. Ceramides are a class of sphingolipids that act as signaling molecules to attenuate cell proliferation, promote apoptosis, and inflammation [4]. In the context of cancer, dysregulation of ceramide metabolism is often observed, which contributes to the uncontrolled growth and survival of cancer cells [5,6]. Understanding the detailed mechanisms of ceramide metabolism in cancer cells can provide insights into the development of new therapies. This review summarizes the multifaceted role of ceramide in cancer cells and identifies strategies for modulating ceramide levels within cells for effective cancer therapy. Furthermore, we discuss various combination therapies, in which ceramide can be synergistically used with other anti-cancer agents, to improve the response rate, and thereby achieve better clinical outcomes in cancer patients. Finally, we highlight several ongoing clinical trials evaluating the potential of ceramide as a new class of anti-cancer therapy.

## 2 Sphingolipid structure and association with pathological states

**Figure 1:**
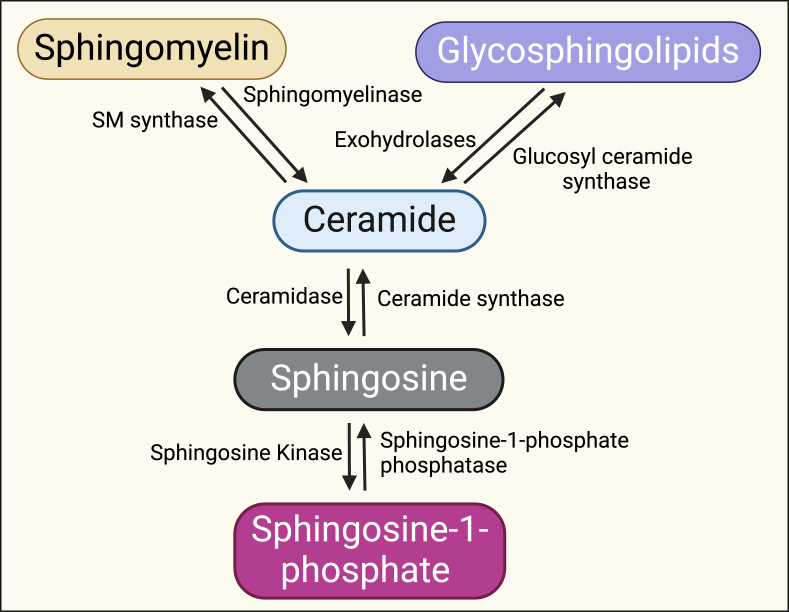
Different classes of sphingolipids and enzymes that regulate their metabolism. There are several classes of sphingolipids including ceramides, glycosphingolipids, sphingomyelins and sphingosine-1-phosphate. The figure also shows the key metabolic enzymes that regulate their interconversion and generation.

Sphingolipids are a class of lipids that have an important role in cell structure, signaling, and various physiological processes [[1], [2], [3]]. The basic structure of a sphingolipid consists of three main components: a sphingoid base, a fatty acid chain, and a head group. There are several classes of sphingolipids including ceramides, glycosphingolipids, sphingomyelins and sphingosine-1-phosphate (Figure 1).

Ceramides are a class of the simplest sphingolipids and consist of a sphingosine backbone linked to a fatty acid through an amide bond [7,8]. The sphingosine backbone is a long-chain amino alcohol that serves as the central structure of ceramide [9] and is derived from the amino acid serine. The nature of the fatty acid chain attached to the sphingosine backbone varies and affects the properties of the ceramide molecule [9]. Ceramides are synthesized by a group of enzymes called ceramide synthases (CerS) [10]. These enzymes catalyze the acylation of sphingoid bases, such as sphingosine, with fatty acids to produce different ceramide species. There are six known CerS in mammalian cells. Each of these CerS displays distinct substrate specificity, tissue distribution, and produces different ceramide species. For example, ceramide synthase 5 and 6 are primarily responsible for producing C16-ceramides, while ceramide synthase 2 and 4 preferentially synthesize very long-chain ceramides, such as C24-ceramides [10].

Ceramide can also be phosphorylated by ceramide kinase to form ceramide-1-phosphate or glycosylated by glucosylceramide synthase to form glycosphingolipids [11]. Glycosphingolipids have sugar residues as head groups and include cerebrosides, globosides, and gangliosides. Gangliosides are particularly abundant in nerve cell membranes and function in cell signaling [11]. Sphingomyelins are made from ceramides through the addition of phosphorylcholine or phosphoethanolamine head groups catalyzed by sphingomyelin synthase. They are predominantly found in cell membranes, particularly in the myelin sheath of nerve cells [11]. Sphingosine-1-phosphate (S1P) is generated when sphingosine is phosphorylated by sphingosine kinases 1 and 2 to form sphingosine-1-phosphate. Sphingosine-1-phosphate is involved in cell signaling and the regulation of various physiological processes [11].

Sphingolipids are involved in various cellular functions, such as cell signaling, cell recognition, and apoptosis [12]. Previous studies have shown that sphingolipids can cause insulin resistance, serve as endocrine mediators, regulate inflammation and immune response, modulate glucose and lipid metabolism, as well as control energy expenditure [2,13]. Therefore, their deregulation is associated with many diseases including neurodegenerative disorders, insulin resistance, type 2 diabetes, sepsis, cancer cachexia, atherosclerosis, and cardiovascular diseases [1]. Since many of the circulating sphingolipids at higher levels have been associated with several human ailments, they may also serve as biomarkers for these diseases. For example, increased levels of circulating ceramides (16:0) or ceramides (24:1) in humans have been observed in ovarian cancer, advanced-stage colorectal cancer, autoimmune diseases such as multiple sclerosis, type 2 diabetes, Alzheimer's disease, sepsis, as well as cardiovascular diseases [[14], [15], [16]]. Similarly, high hexosyl-ceramides (16:0) and hexosyl-ceramides (24:1) levels have also been observed in patients with multiple sclerosis [17]. Additionally, sphingomyelins (16:0), ceramides (16:0), and ceramides (24:1) were strongly associated with cardiovascular diseases driven mortality [18]. Sphingomyelin, ceramides, and hexosyl-ceramides (16:0) and (24:1) are most consistently affected lipid species in case of cancer cachexia and strongly correlate with body weight loss observed in mice and humans with cancer [19].

## 3 Emerging roles of ceramide in cancer cells

**Figure 2:**
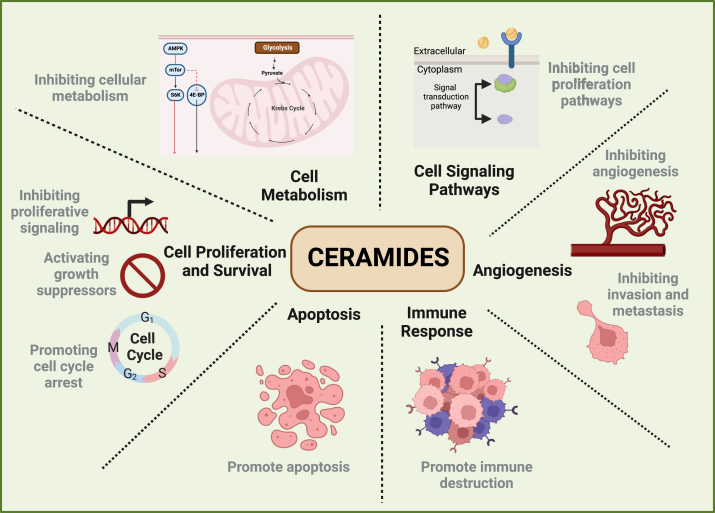
**Diverse roles of ceramide in cancer cells that drive their growth and progression.** Ceramides play several key roles in cancer cells, including the regulation of cellular metabolism, cell proliferation and survival, apoptosis, immune response, angiogenesis and cellular signaling pathways.

Ceramides are important structural components of cell membranes, contributing to cellular integrity and stability [20]. In addition, they have emerged as important signaling molecules that regulate various cellular processes, including cell differentiation, proliferation, apoptosis, and cellular senescence [[21], [22], [23]] (Figure 2). In normal cells, ceramides play a crucial role in maintaining mitochondrial homeostasis, including the regulation of mitochondrial fragmentation and mitophagy [24]. Additionally, ceramides regulate glucose, lipid, amino acid uptake and storage in cells, longevity and senescence, cell differentiation, cilia formation, cell cycle regulation [25,26]. Furthermore, ceramides have also been implicated in regulating whole-body metabolism by modifying intracellular signaling processes, thus emerging as crucial regulators of systemic energy metabolism [27].

In cancer cells, ceramide levels are suppressed, which in turn promote several signaling pathways that in turn promote cancer cell proliferation and survival [28]. They act as second messengers to signal the cells in response to various cellular stresses, such as DNA damage, nutrient deprivation, and oxidative stress [29,30]. Ceramides are shown to influence the activity of protein phosphatases, including protein phosphatase 1 (PP1) and protein phosphatase 2A (PP2A) [31,32], which dephosphorylate key proteins involved in cell cycle regulation and apoptosis [33,34]. Dephosphorylation of PP2A substrates leads to divergent effects, contingent on the specific functions of these substrates. For instance, the dephosphorylation of AKT and MEK results in the suppression of their oncogenic activities. Conversely, the dephosphorylation of the RB protein activates its tumor–suppressive properties, primarily through enhanced binding with the E2F transcription factor, thereby inducing cell cycle arrest [34]. Ceramide in combination with sorafenib synergistically inhibits mitogenic signaling pathways that are involved in promoting cell proliferation. For example, nanoliposomal ceramide in combination with sorafenib inhibited mitogen-activated protein kinase (MAPK) signaling in melanoma and breast cancer cells and also inhibited tumor growth with negligible toxicity [35]. Ceramides also negatively regulate signaling pathways that are activated by growth factors, such as epidermal growth factor and platelet-derived growth factor signaling [36]. Moreover, through the inhibition of AKT, they effectively disrupt the pro-survival phosphatidylinositol 3-kinase (PI3K)/AKT signaling pathway [37], leading to a suppression of cell survival and an augmentation of apoptosis. The inhibition of AKT by ceramide circumvents the suppression of pro-apoptotic factors, thus contributing to a pro-apoptotic environment [38]. Additionally, ceramide has been implicated in modulating the expression and activity of several cell cycle regulatory proteins, such as cyclins and CDKs, which results in cell cycle arrest at different checkpoints affecting proliferation [39]. It is worth noting that ceramides, as signaling molecules, have complex and sometimes opposing roles in cancer [40]. For example, in head and neck squamous cell carcinoma (HNSCC), while ceramide synthase 1–generated C18-ceramide inhibits its tumor growth, ceramide synthase 6–mediated C16-ceramide induces its tumor growth [41]. Moreover, ceramide (Cer) and the precursor of ceramide, which is sphingosine-1-phosphate (S1P) also have opposite functions, and Cer/S1P balance determines the cell fate [42]. Thus, the relationship between ceramides and cancer is complex, and their effect in cancer cells can depend on various factors, including the specific type of ceramide, cellular context, and stage of cancer progression [40,42].

Ceramides are also involved in various metabolic processes, including lipid metabolism, oxidative phosphorylation among others. Ceramides are synthesized *de novo* from fatty acids and sphingosine and this process is regulated by enzymes, such as serine palmitoyltransferase (SPT) [43]. Increased *de novo* ceramide synthesis contributes to inhibition of lipogenesis and synthesis of new fatty acids, which influences the availability of lipids for membrane synthesis and energy storage, thereby reducing cancer cell growth and survival [44]. High ceramide levels also affect mitochondrial membrane integrity and function, resulting in changes to oxidative phosphorylation and energy production. Ceramides further attenuate glycolysis by influencing key glycolytic enzymes and contributing to the Warburg effect [45]. It is shown to inhibit the mammalian target of rapamycin (mTOR) pathway, which is a central regulator of cell growth and metabolism, thus influencing protein synthesis and cellular energy production [46]. Ceramides additionally activate AMPK, which results in metabolic changes, including increased glucose uptake and fatty acid oxidation. Other mechanisms by which ceramide regulate cellular metabolism include inducing ER stress, which affects cell metabolism, protein folding, lipid metabolism, and nutrient sensing [47] and attenuating autophagy, which is also interconnected to metabolism effecting nutrient availability [48]. Thus, ceramides effect overall energy metabolism of cancer cells.

Ceramides are very well known to induce apoptosis, a programmed cell death process that is essential for maintaining tissue homeostasis and eliminating damaged or unwanted cells [49,50]. Some of the mechanisms through which ceramide induces apoptosis are by regulating both mitochondrial and death-receptor-mediated apoptotic pathways. Ceramides promote mitochondrial apoptosis pathway by inducing mitochondrial outer membrane permeability (MOMP). It has been shown that ceramide enhances oligomerization of pro-apoptotic Bcl-2 family proteins, forms ceramide channels, and reduces anti-apoptotic Bcl-2 proteins in the mitochondrial outer membrane [49]. Additionally, ceramide mediated generation of ROS also leads to an increase in oxidative stress promoting the death receptor pathway [51]. Ceramides can also promote apoptosis by activating death receptors on the cell surface, such as the Fas receptor (CD95) or tumor necrosis factor receptor 1 (TNFR1), which leads to the formation of a death-inducing signaling complex (DISC), triggering caspase-8, which results in apoptosis induction. The exact mechanism by which ceramides activate these death receptors can vary depending on the specific context and cell type. However, one common mechanism involves the clustering of death receptors in lipid rafts, which are specialized regions of the plasma membrane enriched in cholesterol and sphingolipids, including ceramides [52]. This clustering can facilitate the recruitment and activation of downstream signaling molecules, ultimately leading to apoptosis [52].

Ceramides play a pivotal role in mediating cellular responses leading to apoptosis and cellular senescence. They induce endoplasmic reticulum (ER) stress, activating the unfolded protein response (UPR) and promoting NASH, which is a condition that predisposes patients for the development of hepatocellular carcinoma, a type of liver cancer [53]. Additionally, ceramides initiate cellular senescence by facilitating retinoblastoma (Rb) hypophosphorylation, resulting in growth arrest [54]. This is further evidenced by studies demonstrating that C6-Ceramide induces G0/G1 cell cycle arrest in WI38 human diploid fibroblasts through enhanced p21 association with CDK2, activating Rb protein and leading to senescence [54]. Ceramides also activate stress-activated protein kinases, including c-JNK and p38 mitogen-activated protein kinase (MAPK), which phosphorylate proteins involved in apoptosis regulation, such as Bcl-2 family members [55]. This phosphorylation disrupts mitochondrial membrane integrity, facilitating cytochrome c release and initiating the intrinsic apoptosis pathway. Moreover, ceramides increase the activation of death receptors like tumor necrosis factor receptor 1 (TNFR1), triggering caspase-8 activation and the extrinsic apoptosis pathway [56]. Despite their role in promoting apoptosis and cellular senescence, the balance between ceramides and other sphingolipids like sphingosine-1-phosphate (S1P), which exert anti-apoptotic effects, is crucial for determining the fate of cancer cells, highlighting the complexity of sphingolipid-mediated cell regulation [5].

Ceramides influence angiogenesis, which is important for tumor growth and metastasis [57]. They have been observed to inhibit angiogenesis by inducing apoptosis in endothelial cells, which are the building blocks of blood vessels. By promoting endothelial cell death, ceramides limit angiogenesis during tumor growth. It also inhibits the migration of endothelial cells, which is an important step in angiogenesis. By inhibiting the ability of endothelial cells to migrate toward angiogenic signals, ceramide negatively regulates blood vessel formation. Vascular endothelial growth factor (VEGF) is an important pro-angiogenic factor. Ceramide interferes with VEGF signaling pathways to suppress the response of endothelial cells to angiogenic stimuli [58]. It also modulates integrin signaling, which affects endothelial cell interactions with the extracellular matrix and other cells [59]. Integrins are cell surface receptors that contribute to angiogenesis by mediating cell adhesion and migration. Thus, ceramide disrupts the formation of endothelial tubes in several ways, which is an important step in the early stages of blood vessel development necessary for angiogenesis. This disruption may have implications for cancer progression.

Ceramides also influence the immune response in the tumor microenvironment, and modulating ceramide levels enhances the effectiveness of immunotherapies [60]. Previous studies have shown that ceramides act as pro-inflammatory mediators by activating inflammatory signaling pathways, such as NF-κB (nuclear factor kappa B) signaling and cytokine production in macrophages. Thus, elevated ceramide levels contribute to the amplification of the inflammatory response [61]. Ceramides are also involved in the activation of T cells, an important component of the adaptive immune system. They modulate signaling pathways that result in T-cell activation and proliferation, and also influence the migration of immune cells by regulating chemotaxis and guiding immune cells to sites of infection or inflammation [62]. Ceramides serve as agonists for Toll-like receptor (TLR) signaling, which is central to the recognition of pathogens by the innate immune system [63]. Thus, the modulation of ceramide levels may impact the tumor microenvironment that determines immune responses and thereby influence tumor initiation and progression.

The specific roles of ceramides in cancer cells vary depending on the tissue type, cancer stage, and other factors. The balance between ceramides and other sphingolipids, such as S1P, adds complexity to their role in cancer etiology and progression. Therefore, future studies are needed to understand the intricate mechanisms that maintain the balance between ceramides and other sphingolipids and develop effective targeted therapies that leverage the regulatory roles of these molecules.

## 4 Modulating ceramide levels in cancer cells for cancer therapy

**Figure 3:**
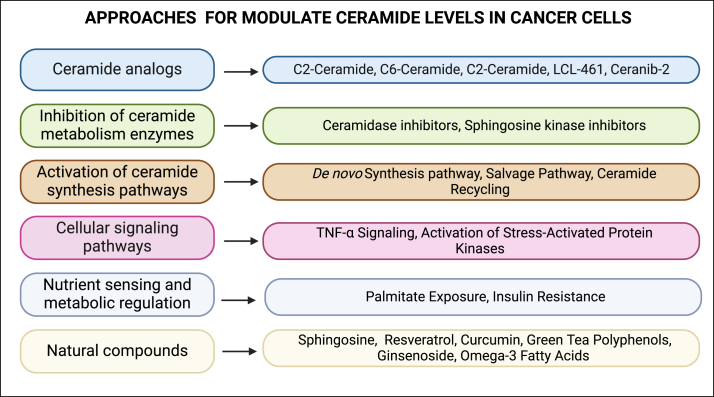
**Approaches and mechanisms that modulate ceramide levels in the cancer cells.** Several different approaches and mechanisms can impact the overall level of ceramides in the cancer cells. These include the usage of synthetic ceramide analogs, inhibition of pathways that metabolize ceramide, activation of pathways that synthesize ceramide among others.

Ceramides have been linked to the response of cancer cells to chemotherapeutic drugs and changes in ceramide levels contribute to drug resistance by regulating apoptosis, cell survival, and DNA repair pathways [13], Furthermore, ceramides have been shown to influence cancer stem cells and their resistance to chemotherapy [23,64]. Thus, modulating ceramide levels or targeting ceramide-related pathways for cancer therapy is an active area of investigation, and several strategies are being explored to modulate ceramide levels in cancer cells. The goal is to either promote ceramide-induced apoptosis or disrupt pro-survival and pro-proliferative signaling that is associated with altered ceramide levels to sensitize cancer cells to chemotherapy. Some approaches by which ceramide levels can be modulated inside the cells include the usage of ceramide analogs, inhibition of pathways that metabolize ceramide, and activation of pathways that synthesize ceramide among others (Figure 3).

Synthetic ceramide analogs have been developed that enhance their bioavailability or specific targeting properties. These analogs mimic the actions of natural ceramides and induce apoptosis in cancer cells [65]. Some analogs also resist degradation, thus prolonging their effects [66]. C2-Ceramide (N-Acetyl-d-sphingosine) is a commonly used analog consisting of a short-chain ceramide with a two-carbon acyl chain. It is a cell-permeable ceramide analog and has been widely used to induce ceramide-mediated responses in various cell types [66]. C6-Ceramide (N-Hexanoyl-d-sphingosine) is another short-chain ceramide analog with a six-carbon acyl chain.

These short-chain ceramide analogs have been extensively utilized in vitro across a variety of cancer cell lines and in vivo models, including those associated with breast, osteosarcoma, and colon cancers, to study the mechanisms underlying ceramide-mediated cellular responses [[67], [68], [69]]. C2-Ceramide and C6-Ceramide are particularly tested for their roles in initiating apoptosis and influencing signaling pathways that regulate cell growth, highlighting their potential as therapeutic agents in cancer treatment [[67], [68], [69]]. Long-chain ceramide analogs, such as C16-Ceramide (N-Palmitoyl-d-sphingosine), more closely resemble the natural ceramides found within cells and have been used both in vitro and in vivo to elucidate their impact on critical cellular processes like cell cycle regulation and apoptosis. Research on C16-Ceramide spans multiple cancer types, including cell line-based in vitro studies for androgen-sensitive prostate cancer, underscoring its potential efficacy for treating prostate cancer with implications for other cancer types [70].

Ceranib-2 is another synthetic ceramide analog that has been shown to induce apoptosis in cancer cells in vitro and has potential for in vivo applications due to its inhibitory effects on tumor growth. Ceranib-2 is specifically studies in the context of breast cancer using in vitro models, and future studies are required to evaluate its in vivo efficacy in suppressing breast tumors in preclinical models [71].

Ceramide levels are regulated by enzymes involved in its synthesis and degradation. Therefore, inhibiting enzymes that promote ceramide degradation can be used as a strategy for treating cancer. For example, activating enzymes, such as sphingomyelinases that convert sphingomyelin to ceramide, or inhibiting ceramidases, which breakdown ceramide, leads to the accumulation of ceramides [72] and triggers apoptosis in cancer cells. One of such inhibitors include N-oleoyl-ethanolamine (NOE). NOE, an inhibitor of acid ceramidase has been used primarily in vitro to promote the accumulation of ceramides and induction of apoptosis in cancer cells and its effects have been studied in head and neck and breast cancer models [73], either alone or in combination with other modalities such as cisplatin in the context of head and neck cancer [74].

Another such drug is SKI-II (Sphingosine Kinase Inhibitor II) and Compound V that inhibit the enzyme sphingosine kinase, involved in the phosphorylation of sphingosine to form sphingosine-1-phosphate (S1P) which functions in contrast to ceramide, promoting cell growth [75]. These inhibitors decrease S1P/ceramide ratio and thereby promote ceramide action. SKI-II has been used in vitro to reduce S1P levels and promote apoptosis, with potential implications for in vivo studies in the context of glioblastoma, gastric cancer and hepatocellular carcinoma [[76], [77], [78]]. Meanwhile, compound V has been tested both in vitro and in vivo breast cancer models [79].

Enhancing the activity of enzymes involved in ceramide synthesis is another strategy to increase cellular ceramide levels. Some of the pathways that activate ceramide synthesis include activation of the *de novo* ceramide synthesis pathways. It has been shown that increased levels or accumulation of intermediates of the ceramide biosynthetic pathway such as sphinganine or sphingosine leads to activation of *de novo* ceramide biosynthesis [80]. Additionally, activation of the salvage ceramide synthesis pathway also contributes to an increase in ceramide levels by activation of sphingomyelinases, an enzyme that hydrolyzes sphingomyelin to release ceramide. Various stimuli, including stress and cytokines, also activate sphingomyelinases [28] to upregulate ceramide levels. Furthermore, alterations of enzymes involved in the recycling of sphingosine and ceramide from complex sphingolipids also contribute to ceramide synthesis. For example, modulation of enzymes such as glycosidases, which hydrolyze glycosphingolipids to generate ceramide, leads to an increase in ceramide levels [81]. Apart from the above-described processes, cellular stress, such as oxidative stress or endoplasmic reticulum stress have also been shown to activate enzymes involved in *de novo* ceramide synthesis. This is mediated through the activation of enzymes, such as serine palmitoyltransferase (SPT), the rate-limiting enzyme in ceramide synthesis [82].

Various signaling pathways also regulate cellular ceramide levels. For example, TNF-α signaling pathway activates ceramide synthesis through various ways, including the activation of the enzyme sphingomyelinase [83]. Activation of cell surface receptors, such as death receptors, also leads to increased ceramide synthesis. For example, the activation of the Fas (CD95) receptor triggers ceramide production either from a neutral sphingomyelinase activity or from *de novo* biosynthesis [84]. Activation of Stress-Activated Protein Kinases (SAPKs) such as c-JNK and p38 MAPK, that are activated under stress conditions also regulate C_16_-ceramide synthesis [85]. Additionally, nutrient sensing and metabolic pathways regulate cellular ceramide levels. For example, exposure to high levels of palmitate (saturated fatty acid and precursor for ceramide synthesis), which is often associated with a high-fat diet, contributes to increased C_16_-ceramide synthesis [86].

Numerous natural compounds, including sphinganine, sphingosine, resveratrol, curcumin, green tea polyphenols like EGCG, certain ginsenosides, and omega-3 fatty acids, have been shown to increase ceramide levels in cells through various mechanisms such as inhibiting ceramide degradation enzymes, enhancing ceramide synthesis, and affecting sphingolipid metabolism [[87], [88], [89], [90], [91], [92]]. These compounds, found in everyday foods and supplements like grapes, red wine, turmeric, green tea, ginseng, fatty fish, flaxseeds, and walnuts, offer therapeutic potential due to their role in ceramide regulation, which can vary depending on cell type, concentration, and experimental conditions. Specifically, curcumin and resveratrol have demonstrated benefits in treating diseases, including anti-tumor effects in glioblastoma and improvements in cardiovascular health, highlighting the medical significance of dietary compounds in modulating ceramide levels and their potential for treatment of cancer and other diseases [93,94]. Nonetheless, it should be noted that the antitumor effects observed from these natural products could be partially due to pathways independent of ceramide level regulation in cancer cells.

To understand the benefit of any therapy it is important to predict its outcome. For ceramide-based therapy there are several ways that could help predict its response. This includes monitoring of some potential biomarkers. For example, measuring changes in ceramide levels during treatment in patients, either in the tumor tissue itself or serum samples could help predict response to ceramide-based therapy. Elevated ceramide levels might indicate a positive response to ceramide-modulating therapies. Besides measuring the ceramide level, monitoring the overall sphingolipid profile could provide a more comprehensive view of the response to ceramide-based therapy [95]. Ceramide is shown to activate the transcriptional activity of p53 [96]. Therefore, monitoring the downstream target of p53 may also help predict the response to ceramide-modulating therapies. Since, ceramide is known to induce DNA damage and activate DNA damage response (DDR) pathways [97], monitoring the markers of DNA damage and repair, such as γ-H2AX and RAD51, may also help predict the response to ceramide-modulating therapies. Additionally, in conditions where ceramide modulating therapies are explored for their anti-inflammatory effects [98], monitoring the markers of inflammation (e.g., cytokines, chemokines) could be relevant. It is important to note that the adaptation of these biomarkers for monitoring ceramide therapy can depend on factors such as the availability of suitable assays for measuring these markers and the resources required for regular monitoring. However, this field is undergoing rapid transformation, and it is will likely become easier to use them on a routine basis. Furthermore, it is important to note that the efficacy of ceramide-modulating therapies can vary depending on the specific disease context and the nature of the therapy. Therefore, disease-specific, and in some cases, the use of multiple biomarkers of response might be needed to accurately predict the response to treatment.

## 5 Combination of ceramide with other therapeutic agents for cancer therapy

**Figure 4:**
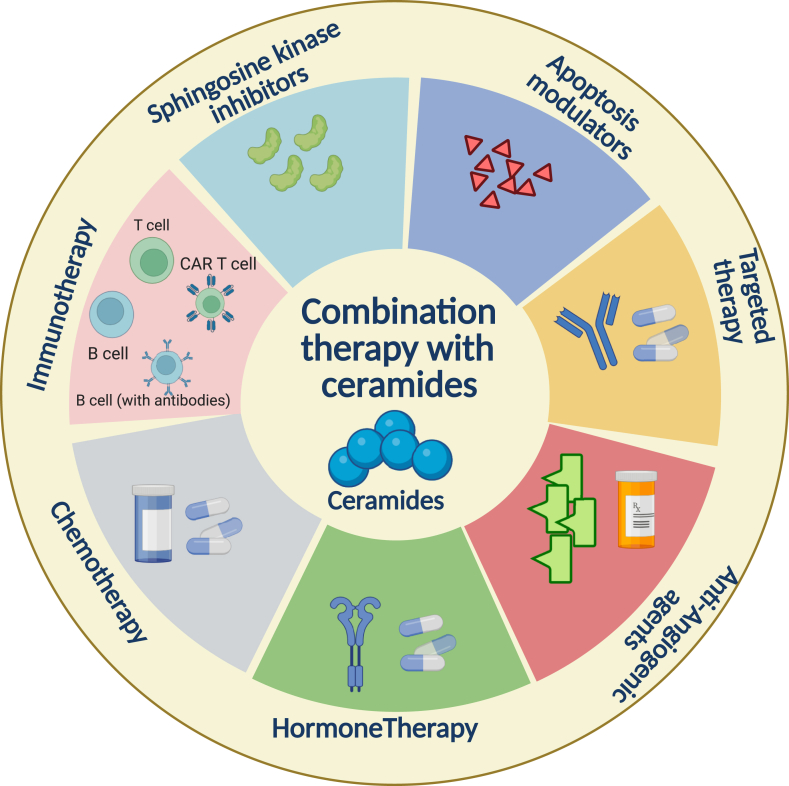
**Ceramide-based combination therapies for cancer treatment.** Ceramide can be combined with several classes of anti-cancer agents such as chemotherapy, immunotherapy, targeted therapies, among others to enhance the therapeutic efficacy of these drugs for treating a wide variety of cancers.

Combination therapy, which involves the use of two or more drugs, is a common strategy to treat cancer in the clinic because of the enhanced effectiveness and potential to circumvent resistance mechanisms compared to single agent-based therapies. When targeting ceramides for cancer therapy, studies have evaluated their combinations with other drugs such as chemotherapeutic compounds, immunotherapeutic agents, apoptosis inducers, among others to achieve synergistic effects (Figure 4).

Previous studies have also shown that combining agents that elevate ceramide levels (e.g., ceramide analogs, sphingomyelinase activators, etc.) with traditional chemotherapeutic drugs, such as doxorubicin, cisplatin, and tamoxifen, enhances apoptosis induction in cancer cells [99,100]. In addition, combining HDAC inhibitors (e.g., vorinostat and trichostatin A) with short-chain C6 ceramide leads to synergistic antitumor effects in in vivo mice xenograft of pancreatic and ovarian cancer models [101]. Combining sphingosine kinase inhibitor, SKI-II, with ABT-263, an inhibitor for anti-apoptotic protein BCL2, has been shown to potently eradicate leukemia cells [102]. Similarly, the sphingosine kinase 1 inhibitor MP-A08 when combined with the BH3 mimetic, Venetoclax also exerts tumor suppressive activity against Acute myeloid leukemia (AML) [103]. Furthermore, Sphingosine kinase (Sphk) inhibitors, such as liposomal safingol, when combined with liposomal C2-ceramide can cause complete remission of AML in a xenograft-based mouse model [104]. The study also showed that the combination treatment of liposomal safingol/C2-ceramide was much more effective in AML cell lines, patient samples and murine xenograft models of AML, as compared to liposomal safingol or liposomal C2-ceramide when used alone [104]. Based on the knowledge that ceramides influence the immune response in the tumor microenvironment, combining nanoliposome C6-ceramide with the anti-CTLA4 antibody have shown improved anti-tumor immunity in hepatocellular cancer [105]. Additionally, a previous study has shown that the effect of hormone therapy, such as tamoxifen, is magnified when used in combination with C6-ceramide [106]. Combining tamoxifen and C6-ceramide led to increased impact on cell cycle progression as well as lysosomal and mitochondrial integrity, which promoted enhanced apoptosis [106]. Ceramide-inducing agents in combination with targeted kinase inhibitors also provides a dual approach to inhibit cancer cell survival and proliferation by targeting two different cancer cell vulnerabilities. For example, combining C6-ceramide with the mTOR inhibitors has been shown to be effective for treating pancreatic and ovarian carcinoma, and C6-ceramide with EGFR inhibitor cetuximab has been shown to be effective for treating KRAS mutant colorectal cancer [101]. Based on the fact that ceramide regulates angiogenesis, it was discovered that the synthetic cell permeable ceramide, N-acetylsphingosine (C2-ceramide) is effective in inhibiting embryonic angiogenesis as well as thalidomide-induced embryonic vascular defect, with an increase in expression of VEGF receptors [107]. However, contrasting resulted were noted when sphingosine-1-phosphate (S1P) was used [107]. Thalidomide is an efficient therapeutic agent used for multiple myeloma and has been thought to exert an antiangiogenic effect. These results underpin that S1P and ceramide might be key to regulating thalidomide-induced antiangiogenic action [107].

Although promising, there are some challenges to ceramide modulation-based therapy. One of the biggest hurdles is the need for selectivity to minimize off-target effects. For example, a study focusing on the inhibition of ceramide synthase 2 within hepatocytes, aimed at decreasing circulating ceramides linked to elevated cardiovascular mortality, demonstrated partial efficacy. While it successfully reduced certain ceramides associated with a higher risk of cardiovascular-related deaths, it concurrently elevated levels of other ceramides, which are positively correlated with cardiovascular mortality [108]. Both ceramide modulating agents - safingol and ABC294640 - have shown to induce some adverse effects on humans in clinical trials [109,110]. The predominant adverse effects observed with Safingol and ABC294640 were generally fatigue, hyponatremia (low sodium levels), reversible hepatic toxicity and gastrointestinal issues, which encompass symptoms like nausea and vomiting [109,110]. Since, safingol is an inhibitor of several enzymes, the off-target effects of safingol could be due to the inhibition of several enzymes and glucose uptake that leads to hepatic toxicity observed in mouse and human studies. As a result, limited number of clinical trials with these agents have been conducted. However, development of nanoparticle-based approaches to selectively deliver ceramide-modulating agents to cancer cells have improved drug delivery and efficacy [111], therefore combining ceramide-loaded nanoparticles with other drugs will offer a novel, selective and effective approach to treating cancer and other diseases. Other approaches by which off -target effects can be tackled includes improving chemical modification of ceramide modulating agents or generating new synthesis agents to increase specificity [11]. Additionally, using a cocktail of multiple inhibitors comprised of ceramide- and sphingosine-metabolizing enzymes with other anti-tumour compounds will also help combat this issue [108]. For example, ABC294640 was reported to have a synergistic effect when used in combination with sorafenib in pancreatic and renal carcinoma cases [112]. Nonetheless, the success of combination therapies depends on several factors, such as the specific cancer type, genetic characteristics of the tumour, and the cancer stage. Therefore, future studies using preclinical models and clinical trials are essential to evaluate the safety and efficacy of these combinations in human patients.

## 6 Clinical use of ceramide for cancer therapy

Clinical trials of ceramide-based therapies are ongoing to evaluate the safety, efficacy, and therapeutic impact of ceramide modulation in cancer patients. Studies are also being conducted to determine the optimal dosage, administration routes, and potential synergies with other cancer drugs. The outcomes of such trials are important for advancing our understanding of the role of ceramide in cancer biology and paving the way for the development of novel ceramide-based interventions that provide novel therapeutic opportunities or enhances the effectiveness of cancer therapy. However, it is worth mentioning that many of the clinical trial are still at the early stages, and therefore, the results and outcomes of these trials are still not fully known. An example of such a clinical trial is using ABC294640, which is a first-in-class orally available inhibitor of the enzyme sphingosine kinase 2 (Sphk2), which promotes autophagy and apoptosis. A Phase I study of ABC294640, in 21 patients with advanced solid tumors (colon/rectal, pancreatic, cholangiocarcinoma, urothelial, and others) has demonstrated that it is tolerated and achieves biologically relevant plasma concentrations [110]. Furthermore, this study also noted that plasma sphingolipid levels could be potentially used as a biomarker for ABC294640-based therapy [110]. Based on the outcome of Phase I trial, a Phase II single-arm trial was conducted both as a standalone intervention and in conjunction with hydroxychloroquine sulfate (ClinicalTrials.gov Identifier: NCT02939807). However, the outcomes of this trial have not yet been disseminated through publication or posting online. Additional trials in cholangiocarcinoma (ClinicalTrials.gov Identifier: NCT3414489) and in metastatic castration-resistant prostate cancer along with androgen antagonists (ClinicalTrials.gov Identifier: NCT04207255) are underway.

Another drug that is being tested in clinical setting for treating cancer is safingol, a saturated derivative of sphingosine. A Phase I clinical trial of safingol, in combination with cisplatin, involving 43 patients with advanced solid tumors, demonstrated that this drug can be safely administered in combination with cisplatin [109]. Notably, one patient with adrenal cortical cancer experienced regression of their liver and liver metastases and another patient exhibited prolonged stable disease [109].

Similarly, BXQ-350, which is a nanovesicle of Saposin C. Saposin C is an allosteric activator for sphingolipid metabolism that lowers systemic S1P and elevates C18 ceramides level [113]. BXQ-350 has been investigated in an open-label multi-center phase I study in children and young adults with relapsed solid tumors [113]. From this study no dose limiting toxicity or BXQ-350-related adverse effects were reported. Notably, one patient with diffuse intrinsic pontine glioma experienced stable disease for five cycles [113]. Currently, additional clinical trials with BXQ-350 for treating different cancers are also ongoing, These trials include newly diagnosed metastatic colorectal cancer (ClinicalTrials.gov Identifier: NCT04207255), children with newly diagnosed diffuse intrinsic pontine glioma or diffuse midline glioma (ClinicalTrials.gov Identifier: NCT04771897).

Based on the observation that introducing ceramides exogenously results in apoptosis induction in cancer cells, several clinical trials are conducted where ceramides are delivered to cancer cells through various methods such as nanoliposomes [114]. In this direction, a Phase II clinical trial was conducted using topical ceramides for breast cancer patients [115]. This trial enrolled 25 patients, and one of these 25 patients showed a partial response with topical ceramide. Although the response rate was not very high, the topical ceramides were well tolerated, as no severe adverse effects were reported [115]. Furthermore, the use of nanoliposome for delivering ceramide inside cells is overcoming the issue with delivering long acyl chain molecules by utilizing short chain ceramide like C6 carried in pegylated nanoliposome [116]. In this regard, ceramide nanoliposome has been tested in a phase I study in patients with advanced solid tumors [117]. For this study, 15 patients with heavily pretreated metastatic disease were enrolled. The results of this study stated that ceramide nanoparticles showed a good safety profile and pharmacokinetics. The study also noted the good potential synergy between ceramide nanoparticles and other anti-cancer therapies [117].

Furthermore, another Phase I study tested the Natural Killer T-cell (NKT) ligand α-galactosyl ceramide (KRN7000) in patients with solid tumors, based on the pre-clinical finding that KRN7000 enhances tumor eradication in mice by activating NKT cells [118,119]. This Phase I study enrolled 24 patients and found that KRN7000 was well tolerated in cancer patients over a wide range of doses [120]. Although no clinical response was observed, however, several patients showed long-lasting stable disease [120]. This study suggested that KRN7000 must be combined with some another therapeutic agent to further enhance its therapeutic efficacy [121]. In this regard, a Phase II study is testing the combination of lenalidomide (orally bioavailable immunomodulatory drug) and autologous mature dendritic cells pulsed with KRN7000 in patients with asymptomatic myeloma (NCT00698776) to determine the optimal dose of lenalidomide for safe usage. Based on the results of this study, a single dose will be selected for the Phase II study of additional patients [121].

The FDA approved Keystone Nano's investigational new drug (IND) application for ceramide nanoliposomes as a treatment for solid tumors on January 5, 2017. This approval was based on the potential of ceramide nanoliposomes to improve cancer treatment through nanotechnology, highlighting the innovative approach of using ceramide integrated into the membrane of a nanoliposome. This method overcomes the challenges of ceramide's insolubility and short half-life in the body, aiming to utilize ceramide's anti-cancer properties effectively. The approval marked the transition to human testing phases, starting with a Phase I clinical trial across multiple sites. Preclinical data indicated potential synergy between Ceramide nanoliposome and multiple systemic therapies, including chemotherapy, targeted therapy, and immunotherapy [106,122]. Collectively, future studies, based on the preclinical findings and the results Phase I and Phase II trial will guide the approval and use of these therapies for treating cancer patients in clinic.

## 7 Limitation of ceramide-based therapy in cancer treatment

The involvement of ceramide in cellular processes associated with cancer growth and progression makes it a promising target for cancer therapy. Despite the potential of ceramide-based therapy in cancer treatment, several challenges must be addressed to optimize its efficacy and safety [123]. One significant hurdle is the complexity of ceramide metabolism, as the interplay between various ceramide species and their regulatory pathways in different cell types can influence treatment outcomes [27]. Achieving targeted delivery of ceramide to cancer cells while minimizing the effects on normal cells also remains a considerable challenge, as its systemic administration may lead to off-target effects and potential in vivo toxicity. Another challenge is the development of delivery systems that can enhance the bioavailability and stability of ceramide [123]. Ceramides are susceptible to degradation, and their efficient delivery to tumor sites requires innovative formulations to overcome biological barriers and improve therapeutic concentrations [123]. Recent attempts to use nanoliposomes as a carrier for ceramide delivery have shown promise in reducing some of these adverse effects [124]. Additionally, developing more specific and effective inhibitors can also help combat current limitations.

The heterogeneity of cancer cells within and between individuals contributes to variable responses to ceramide-based therapy [125]. Hence, tailoring treatments to the specific molecular characteristics of each patient's tumor is challenging and requires a deeper understanding of the intricate signaling pathways. Resistance mechanisms are another major obstacle to the success of ceramide-based therapy [126]. Cancer cells may develop mechanisms to evade ceramide-induced apoptosis, resulting in treatment resistance. Identifying strategies to overcome or prevent resistance is important for the long-term effectiveness of ceramide-based treatments. Finally, our limited understanding of the long-term effects and potential side effects of ceramide-based treatments requires comprehensive preclinical and clinical studies. Rigorous studies into the pharmacokinetics, pharmacodynamics, and potential off-target effects are essential to ensure the safety and tolerability of ceramide therapies in diverse patient populations.

## 8 Conclusion

Ceramide-based therapy holds promise for cancer treatment; however, addressing the challenges associated with specificity, pharmacokinetics, heterogeneity, resistance, and long-term safety is necessary to realize its full potential in clinical applications. Ongoing research efforts are vital to overcoming these hurdles and advancing ceramide-based therapies toward effective and safe cancer treatment.

## Author's contributions

N.W., T.C.B. and R.G. wrote the manuscript and prepared the figures.

## CRediT authorship contribution statement

**Narendra Wajapeyee:** Writing – review & editing, Writing – original draft. **Teresa Chiyanne Beamon:** Writing – review & editing. **Romi Gupta:** Writing – review & editing, Writing – original draft.

## Declaration of competing interest

The authors declare that they have no known competing financial interests or personal relationships that could have appeared to influence the work reported in this paper.

## Data Availability

No data was used for the research described in the article.
